# Evaluating Cancer Care Networks; A Case Study of a Lung Cancer Care Network

**DOI:** 10.34172/ijhpm.2021.98

**Published:** 2021-09-05

**Authors:** Anke Wind, René Limbeek, Henrike Bretveld, Robert van Schijndel, Daan Smits, Wouter de Jong, Hans Smit

**Affiliations:** ^1^Rijnstate, Arnhem, The Netherlands.; ^2^Alliantie Regionale Topzorg (A.R.T.Z.), Arnhem, The Netherlands.; ^3^Netherlands Comprehensive Cancer Organisation (IKNL), Utrecht, The Netherlands.; ^4^Netherlands Cancer Registration, Netherlands Comprehensive Cancer Organisation (IKNL), Utrecht, The Netherlands.; ^5^Slingeland Hospital, Doetinchem, The Netherlands.; ^6^Ziekenhuis Gelderse Vallei, Ede, The Netherlands.

**Keywords:** Cancer Care Networks, Network Evaluation, Quality Improvement

## Abstract

**Background:** Networks are promoted as an organizational form that enables integrated care as well as enhanced patient outcomes. However, implementing networks is complex. It is therefore important to evaluate the quality and effectiveness of networks to ensure it is worth developing and maintaining them. This article describes the development of an evaluation tool for cancer care networks and the results of a pilot study with a regional lung cancer care network.

**Methods:** This study used a combination of qualitative and quantitative evaluation methods. The qualitative evaluation was based on a framework with 10 standards for the organization of an oncological (tumor-specific) care network. Data for the quantitative evaluation were obtained from the Dutch Cancer Registry. The evaluation was performed at a network of three hospitals collaborating in the field of lung oncology.

**Results:** The qualitative evaluation framework consisted of 10 standards/questions which were divided into 38 sub-questions. The evaluation showed that in general patients are satisfied with the collaboration in the network. However, some improvement points were found such as the need for more attention for the implementation and periodic evaluation of a regional care pathway. The start of a regional multidisciplinary meeting has been a major step for improving the collaboration.

**Conclusion:** An evaluation tool for (lung) cancer care networks was successfully developed and piloted within a cancer care network. The tool has proven to be a useful method for evaluating collaboration within an oncological network. It helped network partners to understand what they see as important and allowed them to learn about their program’s dynamics. Improvement opportunities were successfully identified. To keep the tool up to date continuous improvement is needed, following the Plan Do Check Act (PDCA) cycle.

## Background

**Key Messages**

** Implications for policy makers**

- When setting up a network, it is important to also think about how to evaluate them.
- This tool can be used as a baseline measurement when starting a network but more importantly, to evaluate how the network is functioning.
- This tool enables partners in a network to come closer together and define and understand what they see as important.
- This tool can help policy-makers to understand where to direct their resources in order to improve oncology care.

** Implications for the public**

For the public, collaboration within networks is an opportunity to maintain high quality cancer care within the region and close to home. The public should however be able to trust that care is provided according to the highest quality standards and the collaboration and patient transfer within the network takes place optimally. This tool can help the public to know whether the network is functioning according to the highest standards, and if not, that improvements will be made. The proposed method ensures that patients are involved in improving care within the network. Their specific experiences make patients valuable partners in this process.

Networks are widely promoted as an organizational form that enables integrated care as well as enhanced patient outcomes. There are many definitions of networks, but at a minimum a network consists of three or more organizations consciously formed, organized, and directed in to achieve a common goal.^1,2^ The Calman-Hine report published in the United Kingdom in 1995 was the first healthcare policy report to suggest that cancer services should be organized in networks, hierarchized, and integrated to allow better collaboration between health-care providers and co-ordination across settings.^3^ Since then other developed countries have started promoting networks within their national cancer control programs.^4^ Like in other developed countries, in the Netherlands there has been a trend of cancer care network creation. Due to ever rising criteria (for example minimum of cancer surgeries that have to be performed in one year), the Netherlands has seen an increase in concentration of cancer care and mergers of centers.^5^ For high-complex and low-volume tumors in particular, concentration is generally the desired model of care from the perspective of best practice.^5^ However, for high volume tumor care, such as lung cancer, that generally involves palliative care as well, concentration seems to be less favorable. In those cases, the patient seems to benefit more from good tumor-specific networks, in which professionals make mutual agreements about how and where which part of the diagnosis, treatment and aftercare takes place.^5^ In 2014 the Task Force Oncology was established in the Netherlands to continue and build on a cancer care road map. This Task Force has developed a vision with at its core the formation of comprehensive cancer care networks. In other words, partnerships between care providers in the first, second and third lines in the field of cancer care.^6^ One of the outputs of the Task Force is the Course Book for oncological networking.^6^ According to Ferlie et al,^7^ networks should continue to play an important role in cancer service transformation. However, implementing networks is complex because of the number of participants with competing priorities, the multiple levels of governance (national, regional, local), and multiple care processes over a long period.^8^ Questions regarding if or how networks could improve collaboration or the outcomes of care also remain unanswered.^9^ Against this backdrop the importance of evaluating the quality and effectiveness of networks becomes clear.^10^ As far as the authors know, there is no validated evaluation tool for cancer care networks, at least not in the Netherlands. The objectives of this study were therefore (*i*) to develop and pilot a tool for the evaluation of cancer care networks and (*ii*) to evaluate an existing lung cancer network and identify improvement opportunities.

## Methods

###  Evaluation Method 

 There is no clear consensus on which evaluation method is best to be used.^11^ One perspective sees evaluation as objective, structured and relying principally on quantitative approaches.^12,13^ This perspective typically focuses on a program’s outcomes and the extent of its success. Another perspective is collaborative and takes a participatory approach. The goal is to work with stakeholders to understand what they see as important, help them learn about their program’s dynamics and make improvements.^14,15^ This method is highly qualitative. This study applied both types of evaluation.

###  Pilot Setting 

 In the network used in this pilot study, three hospitals collaborate intensively in the field of lung cancer, together with a dedicated radiotherapy center. This tumor specific network is part of a larger organizational network that also includes networks for breast cancer, gastrointestinal cancer and urological cancer. The network was originally established out of the need to collaborate to make sure that at least one hospital in the region would be able to meet the volume criteria (in 2012 these were at least 25 patients treated and a minimum of 20 lung surgeries),^16^ so patients were not forced to be treated outside of their region. Aim was also to improve quality by transparently sharing quality indicators and learning from best practices. The network consists of three general hospitals (hospital A, hospital B and hospital C).

- Hospital A has the following characteristics in terms of diagnostics: all diagnostic options are available like positron emission tomography-computed tomography (PET-CT), endoscopic ultrasound (EUS/EBUS), next generation sequencing (NGS) routine available for 22 genes and when clinically indicated for a ribonucleic acid (RNA) panel, immune histochemistry for anaplastic lymphoma kinase (ALK) and ROS1 is routinely done; in terms of treatment: all treatment options are available including immunotherapy, tyrosine kinase inhibitor (TKI), all surgery and all radiotherapy. Only driver mutations of <5% prevalence are treated elsewhere; in terms of staff: 3 dedicated radiotherapists, 3 dedicated lung (oncology) pulmonologists, 5 dedicated lung surgeons, 4 dedicated thorax radiologists, 2 dedicated lung pathologists, one molecular biologist. The hospital offers supportive and palliative care.
- Hospital B has the following characteristics in terms of diagnostics: all diagnostic possibilities are available like PET-CT, EUS/EBUS, NGS; in terms of treatment: all treatment options are available including immunotherapy, TKI, diagnostic surgery (mediastinoscopy, diagnostic video assisted thoracoscopic surgery) and all radiotherapy. For therapeutic surgery (lobectomy or pneumonectomy) patients are referred to hospital A. Only driver mutations of <5% are treated elsewhere; in terms of staff: 3 dedicated radiotherapists, 3 dedicated lung (oncology) pulmonologists, 1 dedicated lung surgeon, 2 dedicated thorax radiologists, 2 dedicated lung pathologists, 1 molecular biologist (the pathologists and molecular biologist are the same persons as for hospital A and are located at hospital A). All samples are taken at hospital B and transferred for analysis to hospital A. The hospital offers supportive and palliative care.
- Hospital C has the following characteristics in terms of diagnostics: all diagnostic possibilities are available like PET-CT, EUS/EBUS, NGS; in terms of treatment: all treatment options are available including immunotherapy, TKI, diagnostic surgery (mediastinoscopy, diagnostic video assisted thoracoscopic surgery) and all radiotherapy. There is also a dedicated palliative team. Only driver mutations of <5% are treated elsewhere; in terms of staff: 3 dedicated radiotherapists, 5 dedicated lung pulmonologists, 2 dedicated lung surgeon, 2 dedicated thorax radiologists, 2 dedicated lung pathologists, 1 molecular biologist (the pathologists and molecular biologist are the same persons as for hospital A and are located at hospital A). All samples are taken at hospital C and transferred for analysis to hospital A.

###  Evaluation Tool Development 

 The qualitative evaluation was based on a reference framework with 10 standards for the organization of an oncological (tumor-specific) care network. The framework was based on the 21 criteria from the Course Book oncological networking of the Dutch Oncology Taskforce.^6^

 We adapted the Course Book. For example, the following item was removed: “network include at least the most common types of tumor. For rare and complex cancers, the focus is often supraregional or international. In the case of supraregional care, it is relevant to link up as much as possible with networks for nearby care (components)” as this was not applicable in our study which focused on a tumor network. The remaining criteria were combined with existing frameworks within cancer care, such as the quality framework for the organization of cancer care,^17^ the tumor-specific quality framework for lung carcinoma^18^ and the Stichting Oncologische Samenwerking (Oncology Collaboration Foundation) standards.^19^ The resulting framework consisted of 5 domains and 10 standards. For the evaluation of lung cancer care within the regional oncological care network the framework was translated per standard into questions at a general level and more specifically for lung cancer.

 For the quantitative evaluation, in order to gain insight into the effectiveness of the cooperation agreements made, data from the Dutch Cancer Registry (Nederlandse Kankerregistratie, NKR)^20^ were obtained. The NKR is a population-based cancer registry in which trained registry personnel actively collect data from medical records on patient characteristics, such as gender, date of birth, and tumor characteristics, such as the date of diagnosis, tumor type, subsite (according to International Classification of Diseases for Oncology), histologic type, tumor grade, and initial treatment. Stage is recorded according to the Tumor Node Metastasis classification (2010-2016: seventh edition,^21^ 2017: eighth edition^22^). The NKR is maintained by the Netherlands Comprehensive Cancer Organization (Integraal Kankercentrum Nederland, IKNL).^23^

 Together with the partners in the network, IKNL selected a number of indicators with regard to survival, diagnostics, treatment patterns and referral patterns regarding lung cancer care that were available in the NKR up to and including 2017.

###  Evaluation 

 The evaluation took place in 4 phases: (1) Document review; (2) Interviews and survey; (3) Quantitative data collection; (4) Analysis.

####  1. Document Review

 We looked at documents in which the cooperation agreements made were recorded. These included a covenant in which the cooperation was established, policy and vision documents, (transmural) care pathways, service level agreements, minutes of policy and substantial consultation. Based on the documents, it was examined for each standard which agreements were made at the start of the care network.

####  2. Interviews and Survey

 (a) Medical and nursing specialists from the centers of the network involved in lung cancer care. These interviews took place between April and June 2019. Most medical and nursing disciplines involved in lung cancer care were informed in advance about the objectives of the evaluation. In consultation, a discussion partner was selected for each discipline and center and approached for an interview. A choice was then made per discipline between several individual interviews or one group interview. A questionnaire was drawn up for each discipline based on relevant topics from the framework. Interviews were performed on location and by phone. A report was written for every interview.

 (b) Patients who have received care in more than one of the network centers. A sample of 11 patients was interviewed. The group of patients who were eligible for an interview visited at least two centers of the network for diagnostics and/or treatment. The selection was made with the help of the nursing specialists from the network centers. Patients were asked on a regular visit to the outpatient clinic whether they were willing to participate. A written statement of consent was signed. Prior to the interview, patients received additional information by phone and a written document about the objective and nature of the project. For the patient questionnaire, topics in which the patient had relevant experience were selected from the framework, with a focus on transfer moments. A report was written per interview.

 (c) Directors, managers and support staff of the network. A selection was made of people who are involved in the network as director, manager/director or quality advisor. With regard to the directors a group interview was chosen, the other people were interviewed individually. The focus of the questions was on the standards from the framework that related to management and policy.

 (d) Survey to general practitioners (GPs). In the selection of GPs, it was decided to approach the GPs of the patients interviewed because there was a direct involvement. During the interviews with the patients, the name of their GP was asked and as well as permission to approach the GP. GPs proved difficult to approach and there was little enthusiasm for interviews, so it was decided to write a short questionnaire (Supplementary file 1) via email, with a focus on communication with the patient and other care providers and providing information about the patient.

####  3. Quantitative Data

 The selected indicators were compared over three different time periods: two years before the start of the network (2012/2013), two years after the start of the network (2014/2015) and the next two years in which the cooperation was developed further (2016/2017).

 Per period, both the data of the three separate settings as well as the combined average of the network was shown. To put the data in perspective and to be able to benchmark, data from the rest of the Netherlands were also shown.

####  4. Analysis

 Two methods were used to analyze the qualitative and quantitative data.

####  Qualitative Data Analysis

 A deductive form of the qualitative content analysis^24^ was used to analyze the qualitative data retrieved through the interviews and the survey. This method contains 8 steps: (1) Read through the interview transcripts and make notes; (2) Go through the notes and list the different types of information found; (3) Read through the list and categorize each item (domains of the framework were used as main categories); (4) Repeat the first three stages for each interview transcript; (5) Collect all of the categories or themes and examine each in detail, considering its fit and its relevance; (6) Categorize all data (all transcripts together) into minor and major categories/themes; (7) Review all categories and ascertain whether some categories can be merged or sub-categorized; and (8) Return to original transcripts and ensure that all the information has been categorized.

####  Quantitative Data Analysis

 All patients with lung cancer diagnosed in one of the hospitals of the network between 2012 and 2017 were selected from the Netherlands Cancer Registry. Numbers of patients were reported per hospital, specifying the pathological characteristics and the proportion of patients with non-small cell lung cancer (NSCLC) stage IV for which PD-L1 (Programmed death-ligand 1) was determined, excluding patients with epidermal growth factor receptor (EGFR) mutation or ALK translocation. Treatment patterns were analyzed according to hospital, period, histology and stage, as well as referral patterns.

 Information on the vital status of the patients was obtained from the population registries network, which provides virtually complete coverage of all deceased citizens of The Netherlands. The follow-up data was complete up to February 1, 2019. Overall survival and the corresponding 95% confidence intervals were calculated per hospital, period, for NSCLC, including patients without pathology verification, and small cell lung cancer and stage, only in case of more than 10 patients per stratum. Cox regression analyses were performed to calculate the hazard ratios, univariable and multivariable additionally adjusted for age and gender. The analyses were performed using SAS version 9.4 (SAS Institute, Cary, NC).

## Results

###  Evaluation Tool

 For the qualitative evaluation, a reference framework with 10 standards was developed that is specifically aimed at the organization of and cooperation within cancer care networks, especially for lung cancer care. To perform the interviews the 10 standards were translated into 10 questions divided into 35 sub-questions. Table 1 gives an overview of the standards and questions used in this study.

**Table 1:** Evaluation Framework

**Policy Area**	**Criteria **	**Standards**	**Questions**
Management - Policy	Optimal composition (University Medical Center, top clinical, general hospitals, 1st, 3rd line)	1. There is a regional partnership in which all relevant chain partners are involved and all diseases within the tumor type can be treated optimally	Central: Does the partnership (together with chain partners) function as a comprehensive cancer network for lung cancer care in the region? Sub: 1. Is there an optimal composition of the partnership with representation from a University Medical Center, top clinical, general hospitals, 1st and 3rd line? 2. Are agreements made about the management of the partnership, both in terms of policy and content? 3. How is collaboration with 1st line care providers (GPs) organized? 4. What has been agreed with regard to possibilities for diagnosis and treatment at the various chain partners and referral within and outside the region? 5. Are there admission criteria for participation in the partnership? 6. Are agreements made about the conditions under which the partnership can be abandoned?
	Administrative agreements, governance and evaluations	2. Collaboration agreements in the chain are fixed at administrative level and are elaborated in service level agreements 3. The input of the patient's perspective is guaranteed when drafting collaboration agreements in the chain	Central: Are regional collaboration agreements for lung cancer care fixed at administrative level? Sub: 7. Have agreements been made within the partnership on the availability and accessibility of specific care, guaranteeing high quality, guaranteeing interaction between care, research, innovation, knowledge and financing? 8. Has there been input from a patient perspective when making collaboration agreements? 9. Are the agreements made complied with according to the various parties involved? 10. Are the agreements made periodically evaluated?
Patient care	Regional /transmural care paths	4. There is an implemented transmural care path	Central: Have cooperation agreements on tactical level regarding lung cancer care been recorded both within the partnership and with chain partners? Sub: 11. Is there a regional/transmural care path that states who undertakes which activities at which stage for patients with (suspected) lung cancer within the care chain? 12. Are agreements made on a clear point of contact (case manager/main practitioner) for patients with (suspected) lung cancer throughout the chain? 13. Are agreements made both within the partnership and with chain partners about the provision of palliative care? 14. Are the agreements made complied with according to the various parties involved? 15. Are the agreements made periodically evaluated?
	Multidisciplinary meetings: all patients are discussed; creating echelons (general, region, expert multidisciplinary meetings)	5. All patients with (suspected) lung cancer are discussed in the multidisciplinary meetings according to the applicable criteria^19^ (All patients need to be discussed pre-treatment. The following disciplines have to be present during the Multidisciplinary Meeting: pulmonologist, (lung and/or thoracic) surgeon, radiation-oncologist, radiologist/nuclear medicine physician, pathologist, case manager and/or oncology nurse and/or oncology nurse specialist)	Central: Are all patients with (suspected) lung cancer within the partnership (the partners with the network) discussed in multidisciplinary meetings? Sub: 16. Are agreements made, both within the partnership and with chain partners (first line partners and academic hospitals), regarding the structure of meetings/creating echelons for multidisciplinary meetings? 17. Are agreements made within the partnership regarding reporting, decision-making, feedback and boundary conditions at multidisciplinary meetings and do they meet the relevant criteria? 18. Are the agreements made complied with according to the various parties involved? 19. Are the agreements made periodically evaluated?
	Care plans for patients Shared decision making	6. Agreements have been established on the preparation of a (after) care/treatment plan for patients with (suspected) lung cancer	Central: Are agreements made about the preparation of a (after) care/treatment plan for patients with (suspected) lung cancer? Sub: 20. Is attention paid when drawing up a care/treatment plan to: • shared decision-making • accessibility therapies • advanced care planning 21. Are the agreements made complied with according to the various parties involved? 22. Are the agreements made periodically evaluated?
	Communication, information, transfer and data exchange	7. Agreements have been made by the chain partners on communication and information which is provided to patients, as well as mutual communication with regard to patients with (suspected) lung cancer	Central: Are agreements made, both within the partnership and with chain partners, on communication and information which is provided to patients, as well as mutual communication with regard to patients with (suspected) lung cancer? Sub: 23. Are agreements made, both within the partnership and with chain partners, regarding the use of media/infrastructure and mutual data exchange? 24. Are agreements made with fellow care providers and chain partners about timely informing about the care/treatment plan and the care process in which the patient is located? 25. Are the agreements made complied with according to the various parties involved? 26. Are the agreements made periodically evaluated?
Clinical studies/trials	Participation and contributions to research agenda	8. Agreements have been made by the chain partners regarding participation in clinical studies/trials for lung cancer care	Central: Are agreements made, both within the partnership and with chain partners, about participation in clinical studies/trials with regard to lung cancer care? Sub: 23. Are the agreements made complied with according to the various parties involved? 24. Are the agreements made periodically evaluated?
Knowledge sharing	Knowledge sharing; appoint leading centers per tumor type	9. Agreements have been made by the chain partners about knowledge sharing and the promotion of expertise with regard to lung cancer care	Central: Are agreements made, both within the partnership and with chain partners, about sharing knowledge and promoting expertise with regard to lung cancer care? Sub: 29. Are the agreements made complied with according to the various parties involved? 30. Are the agreements made periodically evaluated?
Quality assurance	Continuous improvements with PDCA, evaluations, benchmarks; transparency Participation in registrations, audits; Minimize unwanted variation	10. Agreements have been made by the chain partners on quality policy with regard to lung cancer care	Central: Are agreements made on quality policy with regard to lung cancer care, both within the partnership and with chain partners? Sub: 31. Are agreements made on continuous improvement (PDCA), evaluations, certification policy, benchmarks, and transparency? 32. Are agreements made about participation in registrations for NKR, PALGA and DLCA, registration of expensive medicines and registration of PREMs and PROMs? 33. Are agreements made about minimizing unwanted variation? 34. Are the agreements made complied with according to the various parties involved? 35. Are the agreements made periodically evaluated?

Abbreviations: GPs, general practitioners; PALGA, Pathologisch-Anatomisch Landelijk Geautomatiseerd Archief (Pathological-Anatomical Countrywide Automated Archive); DLCA, Dutch Lung Cancer Audit; NKR, Nederlandse Kankerregistratie; PREMs, Patient Reported Experience Measures; PROMs, Patient Reported Outcome Measures; PDCA, Plan Do Check Act.

###  Interviews

 The performances of the network varied on the different standards, of which a selection is shown in Table 2. Organizations within the network are portrayed anonymously. The results are structured according to the domains of the interview framework. Based on the evaluation several improvement suggestions were made by the evaluation team (who performed the evaluation). These suggestions were discussed by the Regional Multidisciplinary Team (containing health professionals from all three hospitals) and approved. Table 2 shows a selection of these improvement suggestions.

**Table 2:** Interview Outcomes and Improvement Suggestions

**Domain**	**Standard**	**Outcome Interviews**	**Improvement Suggestions**
1. Management and policy	Collaboration agreements in the chain are fixed at administrative level and are elaborated in SLAs	The regional partnership is limited to collaboration agreements between three regional hospitals and the radiotherapy center. Most care providers see themselves as part of their own hospital first and part of the regional partnership second. The regional multidisciplinary meetings stimulate the awareness of being part of the network. The collaboration between the partnership and the primary care providers is organized per hospital.	
	There is a regional partnership in which all relevant chain partners are involved and all diseases within the tumor type can be treated optimally	The network made reference agreements per tumor type for collaboration with a preferred reference center. The degree of collaboration is determined for each tumor type and is partly dependent on the diagnostic and treatment options within the partnership. Looking specifically at lung cancer it was found that there are no official cooperation agreements with an academic partner. But there is an informal collaboration with a University Medical Center.	
	Collaboration agreements in the chain are fixed at administrative level and are elaborated in SLAs	Policy is developed within the steering committee of the network together with the different Tumor Working Groups, supported by a program office.	
2. Patient care	There is an implemented transmural care path	At the start of the network collaboration, coordination of the processes surrounding lung cancer care took place. This has not yet led to an established transmural care pathway and there is no periodic evaluation and adjustment of the care pathway. Patients reportedly feel that transfers between the different hospitals are organized well.	The agreements and the overall working method, as described in the care pathway need to be effectively implemented on a regional basis. It is advised that the care pathway should be properly assessed and put on the agenda by network partners. The care pathway can be used in the different hospitals as a basis for establishing SLAs and referral method with support services. In order to ensure that the care pathway remains up to date, it should be periodically (at least once a year) evaluated and adjusted (PDCA). Currently the accountability for pathways lies with the regional tumour board, which regularly meet. Each meeting a part of the pathways is discussed and updated if necessary updated.
	All patients with (suspected) lung cancer are discussed in the multidisciplinary meetings according to the applicable criteria	Multidisciplinary meetings take place in a regional context in which all patients are discussed since 2018. According to the medical and nursing specialists involved, regional consultation leads to better coordination in the treatment and follow-up processes. More mutual involvement between the participants from the different centers is also established. The interviewees also indicated that the quality of their care and outcomes for patients improved because of the RMDM. Some interviewees however indicated that the quality of the RMDM could be improved.	With regard to the organization of the regional multidisciplinary meetings, improvements can be made in efficiency, the structural participation of all relevant disciplines, the available technical facilities, coordination with a reference center and the possible inclusion of patients in ongoing clinical studies.
	Agreements have been made by the chain partners on communication and information which is provided to patients, as well as mutual communication with regard to patients with (suspected) lung cancer	The treatment policy that has been discussed multidisciplinary regionally is recorded and is clear to patients. Patients indicate however that they feel unprepared for the change in scale and atmosphere when referred to another center within the network.	
	Agreements have been established on the preparation of a (after) care/treatment plan for patients with (suspected) lung cancer	Healthcare providers have limited access to the electronic patient record elsewhere if the patient has been referred from or to another center within network.	
Clinical studies / Trials	Agreements have been made by the chain partners regarding participation in clinical studies/trials for lung cancer care	The evaluation showed that very few patients are referred for studies and no joint research policies are written. The centers within the network have the ambition to collaborate in clinical studies.	It is recommended to start with drawing up a joint vision on research/participation in clinical studies, also with regard to referral outside the network. Subsequently, it is recommended to develop a construction in order to set up facilitate joint studies, for example by centrally arranging statements from the Medical Ethics Review Committee and other steps at initiating research.
Knowledge sharing	Agreements have been made by the chain partners about knowledge sharing and the promotion of expertise with regard to lung cancer care	There is no formal knowledge sharing between the partners in the network. The different disciplines attend the national/regional meetings.	Network multidisciplinary knowledge sharing meetings improve the exchange of knowledge between the participants from the different centers. The input from these meetings can also be used to update the care pathway. To avoid having to reinvent the wheel every time new techniques are introduced, more knowledge sharing should take place.
Quality assurance	Agreements have been made by the chain partners on quality policy with regard to lung cancer care	Management and healthcare providers within the network have sufficient attention to relevant quality indicators. However, improvement initiatives are often not shared and agreements often do not go through the PDCA^25^ quality cycle. Service level agreements have not been established for with all supportive services.	Across the board, attention to securing the agreements made by going through the quality cycle is a point of attention. It is desirable that improvement initiatives are shared and coordinated within the network.

Abbreviations: PDCA, Plan Do Check Act; RMDM, Regional Multidisciplinary Meeting; SLAs, service level agreements.

###  NKR Data

 In this paragraph a short summary is given of the findings from the NKR data analysis. Table 3 shows an overview of the number of patients per stage in the network compared to the national numbers.

**Table 3:** Absolute Number of Patients, (for the Network Also Percentage of Patients out of National Number of Patients) Diagnosed With NSCLC

	**2012-2013**		**2014-2015**		**2016-2017**	
	**Network**	**National**	**Network**	**National**	**Network**	**National**
Stage I	187 (4.8%)	3902	205 (4.6%)	4476	217 (4.4%)	4883
Stage II	93 (5.4%)	1731	102 (5.4%)	1875	98 (4.7%)	2071
Stage III	230 (4.9%)	4700	230 (4.9%)	4707	230 (4.8%)	4765
Stage IV	484 (4.8%)	10 165	513 (4.8%)	10 653	509 (4.6%)	11 037
Total	994 (4.8%)	20 498	1050 (4.8)	21 711	1054 (4.6%)	22 756

Abbreviation: NSCLC: non-small cell lung cancer.

####  Survival

**Figure 1:**
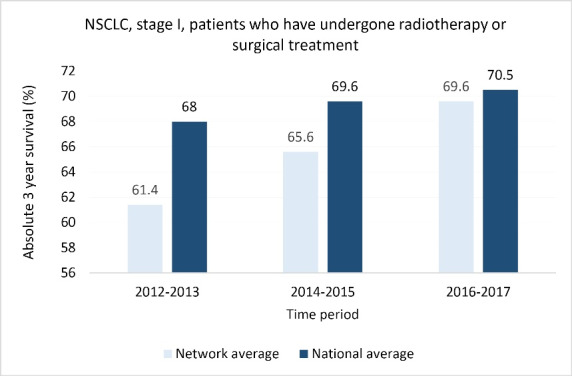
Absolute 3 Year Survival NSCLC, Clinical Stage I, Patients Who Have Undergone Radiotherapy or Surgical Treatment. Abbreviation: NSCLC, non-small cell lung cancer.

 For NSCLC, clinical stage I patients who have undergone radiotherapy or surgical treatment, the absolute 3-year survival within the network centers together has increased towards the national average, but is not yet at the same level (see Figure 1).

####  Diagnostics

 The proportion of tumors with NSCLC, clinical stage IV, excluding patients with EGFR/ALK mutation, where PD-L1 measurement was performed in the 2017 period (49%, N = 117), is considerably higher than the national average (32%, N = 1420).

####  Reference Patterns

**Figure 2:**
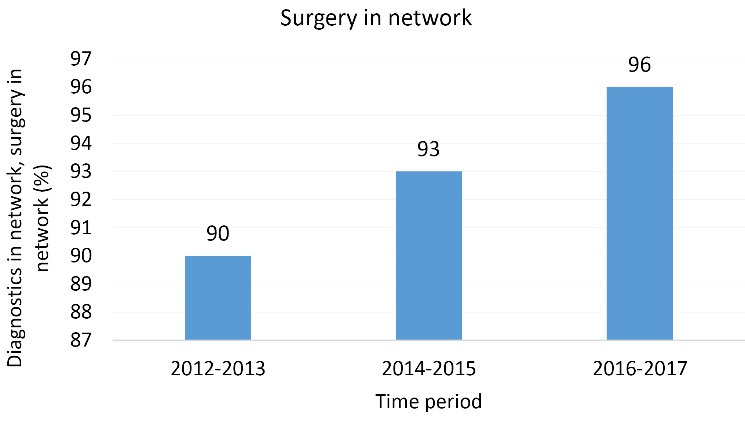
Percentage of Lung Cancer Patients Diagnosed in the Network That Have Surgery in the Network. 2012-2013 N = 179; 2014-2015 N = 235; 2016-2017 N = 227.

 Since the collaboration within the network started, fewer patients have been referred elsewhere for surgery (see Figure 2). Network agreements ensure more retention of patients in the region.

 However, the referral for surgical treatment within the network has slowed (patients stay in their “original” hospital) during the years following the creation of the network. Only patients from hospital B (a hospital without therapeutic lung surgery) referred patients to the other hospitals. Hospitals A and C did not refer surgery patients to each other. There also appears to be little to no referral for non-surgical treatment (as primary treatment) to a network partner. It also seems that patients are very limitedly referred for, for example, participation in clinical trials. In general, hospital A and B have trials for all stages of lung cancer in which they recruit their own patients. In hospital A there are in general 5 open trials a year, in hospital B 3. However, hospital A and B rarely included patients from their network partners.

 Although within the network there are agreements about cooperation with one expertise center per tumor type, looking at lung cancer two out of the three network centers (hospital B and C) show a decreased referral rate to this specific expertise center. The reference to other centers (elsewhere) increased for both hospitals during this period. See Supplementary file 2 for more information on referral patterns.

## Discussion

 In this study, we developed a tool to evaluate cancer care networks consisting of 10 standards. The tool was successfully tested in a lung cancer network of three hospitals in the Netherlands to assess its suitability for evaluating the network and yielding improvement suggestions. The network offers all aspect of the cancer care pathway. Within the network all types of treatment are offered, only driver mutations of <5% are treated elsewhere. All three hospitals use the same pathological lab with fully equipped NGS possibilities. Molecular diagnostics are state of the art with NGS routine available for 22 genes and when clinically indicated for an RNA panel. Immune histochemistry for ALK and ROS1 is routinely done. Palliative care, supportive care, and survivorship care are present at all 3 hospitals. Looking at the reason the network was established in the first place, which was to maintain sufficient patient numbers and optimal quality of care so patients can stay in their own region for treatment, we found that the collaboration within the network has indeed caused fewer patients to be referred elsewhere for surgery. A large survey amongst Dutch cancer patients^26^ showed that 55% of the patients wanted to stay close to home for their treatment. The other 45% was willing to travel without maximum travel time, but only if that was necessary to get specialized care. This enforces the rationale for establishing the network, meeting the patient wish to receive specialized care close to home.

 The basis of collaboration was trust. The trust that all three participating hospitals would not be threatened in the way they could serve and treat the majority of their own patients. The trust is built on the fact that very limited patient journey is shared. Only when therapies are not available in one hospital, the patient is transferred to another. In terms of diagnostics the same pattern is present. In times of shortage (delay), the patient is asked to go to one of the partners. Because there is no financial penalty we believe that the “trust” is a true condition. Issues damaging this trust could however arise in the coming years such as the feeling of a threatened clinical autonomy; developments in financial systems that could lead to fear of loss of reimbursement income.^27^

 With regard to the second objective, which is sharing quality indicators and learning from best practices, management and healthcare providers share and discuss quality indicators openly within the network. Whether this has led to quality improvements that otherwise would not have been achieved remains unknown. The most important tool for collaboration according to the interviewees, the Regional Multidisciplinary Meeting (RMDM) was not in use until 2018.

 In general, the evaluation standards revealed that the network is on the path to become well-organized. Patients generally feel that the transfer between different hospitals in the network is organized well. This is mostly due to the extensive coordination of the processes within the care pathway at the start of the network. This care pathway was, however, never officially formalized and has not been updated since it was developed. With the rapid development in diagnostic and treatment options this means that there is a risk that the care pathway will become outdated and no longer useful. Nevertheless the three partners kept on innovating according to the national guidelines and international medical progress. Accountability for pathways lies with the regional tumor board, who made a plan for updating the pathway regularly. One of the biggest improvements of working in the network according to the interviewed professionals was the development of the RMDM. The interviewees felt that the RMDM led to better coordination in the treatment and follow-up process and improved quality of care. The evaluation however showed some improvement points, such as technical facilities, efficiency – especially meeting discipline or insufficient documentation of the diagnostic workup – and the structural participation of all relevant disciplines. With regard to the last two improvement points, echeloning of the multidisciplinary meeting may be helpful. Echeloning refers to the grouping of, in this case, patients with a similar profile into one echelon.^28^ The more complex echelons require more expertise and the Multidisciplinary Meeting will require the attendance of more multidisciplinary team members.^26^ In practice the majority of cancer patients are part of the lowest echelon, low complexity patients, which require fewer Multidisciplinary Meeting members.^27^ Workload per discipline could be reduced and the possibility of structural participation of all relevant disciplines would increase.

 Whether the RMDM actually resulted in more uniformity within the network with regard to the different treatment patterns and higher quality could not be analyzed based on the NKR data, as data for 2018 were not yet available at the time of the evaluation. The current impression however is that since 2018 the uniformity has significantly improved.

 Although there is an ambition to have more patients participate in clinical studies, hardly any results have been achieved through cooperation within the network. Making agreements and increasing support for participation in studies is recommended, as well as closer cooperation with a reference center. Center A and B already participate in many of the same national trials. Making clinical trial participation a regular topic at the RMDM could contribute to the ambition to include more patients. More formal collaboration with a center of expertise (University Medical Center) with regard to patient care, in particular with rare and complex disorders, and with regard to clinical studies/trials and translational research, could further improve regional lung cancer care.

 Although it is favorable that patients can be treated in their own region, especially with regard to palliative care, the need to maintain sufficient patient numbers could lead to patients not always receiving appropriate/optimal treatment. Volume pressure could lead to hospitals opting for surgery more often where another treatment regimens might be more appropriate. It is important that the network is conscious of the risk to prevent this from happening.

###  Limitations

 A possible limitation of this study is the Course book^6^ used as a basis for the interview framework, as it did not fully fit the purpose of this evaluation. The Course book focuses on care networks that work as a comprehensive care network (this always includes an academic partner). This disqualifies more small-scale healthcare networks. The most important thing is to check whether all relevant chain partners are involved in making cooperation agreements that are in line with the objective of the care network. It might not be necessary to include an academic partner within the network to ensure access to academic care. Good cooperation agreements might suffice.

 The standards did not include a question about the structure of the partnership. The evaluation of the lung cancer network showed that in particular, agreements must be made about this structure (the what-question), whereby a distinction is made between: (1) policy-based management, (2) substantive management and (3) implementation/operational matters. Due to lack of data we did not include some quality standards that ideally would have been included such as waiting times.

 Future network standards should focus on “connectivity” within the network. It should include that those standards notionally sit “on top of” quality standards regarding the diagnosis and treatment in individual hospitals. Although this means that we do not regard a network as a proxy for a hospital we recommend to include more quality indicators such as: Waiting Times (as a proxy for Outcomes, and tracking of continuity of care); Percentage deviations from Clinical Guidelines; Percentage of patients referred to Clinical Trials to network partner (this is already known for the individual hospitals in this study).

 For this study, with regard to primary care network partners, we only focused on GPs. Due to the low response of GPs it was difficult to take their perspective of the network into account. GPs banded together and selectively leavened their contributions because they were inundated with all kinds of questionnaires. The average number of cancer patients per year that a GP sees is low. We tried to circumvent this by specifically approaching those GPs of whom we had already spoken to a patient, but this did not lead to more willingness to be interviewed. The limited response gives reason to reconsider how the (regional) GP can best be involved in such an evaluation process.

 When analyzing the NKR data, the often small numbers in the individual network centers should be taken into account. Therefore, far-reaching conclusions cannot generally be reached. It is not always clear to what extent agreements made in the network have led to differences in data over the different periods. Nevertheless, the data show a clear indication of the direction in which the network is moving and how it compares to the rest of the Netherlands.

 Finally, the question is whether the improvements found are due to the network. Some improvements may have taken place anyway, there is no clinical setting to correct for this. No clear outcome indicators with regard to cancer network effectiveness have been established yet. This enforces our decision to use both an objective, structured and quantitative approach as well as a qualitative participatory approach. When little objective and structured outcomes are available, opinions of stakeholders, eg, participants, sometimes give the best insight into the outcomes of, in this study, the implementation of a cancer care network.

###  Lessons Learned

 Multiple lessons were learned during the pilot of the evaluation tool. Firstly the evaluation showed it is not necessary for every discipline to interview someone from every center in order to get a complete picture of the situation. It seems that most of the time one representative per discipline would be sufficient. Based on the information gathered in advance, one could make a better estimate of the added value per interview.

 The choice between an individual and a group interview depends on various factors, such as the time investment. Another factor that needs to be taken into account is the extent to which “sensitive” topics are raised, causing people to be less likely to express themselves or respond critically in the presence of colleagues from other centers. To what extent can people complement each other during the interview is also important to assess when choosing between an individual or group interview.

 Because of the planning, time investment and depth, one can choose to conduct the evaluation in phases and limit a phase to one or a limited number of preselected standards or components of the care process.

###  Continuous Improvement

 The evaluation tool itself needs continuous improvement as data come back from piloting in networks themselves. Learnings from the evaluation should be fed back into the standards to define clearer standards or indicators (sharpening up on the “are agreements made” formula). So, if the evaluation shows that a point, like the lack of being able to access an Electronic Health Record within the network, is an issue that could become a future defined standard (sharpening up sub 23 of the tool used in this study). Similarly with RMDMs, as these are concluded by clinicians to be improving effectiveness; tracking the number and percentage of patients discussed in the RMDM could become a new quantitative indicator. Ensuring this evaluation process itself can be subject to the Plan Do Check Act (PDCA) cycle.

## Conclusion

 The evaluation tool has proven to be a useful method for evaluating collaboration within an oncological network. It helps network partners and stakeholders to understand what they see as important, as well as help them learn about their program’s dynamics and possible improvements.

 The pilot has shown that in many areas the collaboration between the network partners with regard to lung cancer care has clearly taken shape since its inception, despite many agreements not being formally established and structurally evaluated. Patients were generally satisfied with the cooperation and information provision during transfer moments between the network partners. The healthcare professionals involved were also satisfied, but often maintained a hospital minded focus instead of thinking as a network. The RMDMs, started in 2018, have given a new important and essential impulse to the collaboration and strengthened the network thinking. The most important improvement opportunities are mainly to make concrete agreements about a transmural care pathway, RMDM, clinical studies and to implement and record these. To keep the tool up to date there is a need for continuous improvement of these evaluation standards. This requires feeding back data from networks, so that standards become sharper, and the number of quantitative process measures can be increased.

## Acknowledgements

 The authors would like to thank all network members that participated in the interviews and/or provided documented information. Special thanks to the patients who, despite their illness, took the time to tell us what they think of the network, especially what is going well, but also what could be improved.

## Ethical issues

 Ethical approval was deemed unnecessary as there was no medical scientific research and patients were not subjected to actions or imposed rules of conduct.

## Competing interests

 Authors declare that they have no competing interests.

## Authors’ contributions

 AW designed the study, acquired the data, analysed and interpreted the data and drafted the manuscript. RL designed the study, acquired the data, analysed and interpreted the data and drafted the manuscript. HB analysed and interpreted the data, performed the statistical analysis and critically revised the manuscript. RvS critically revised the manuscript. DS critically revised the manuscript. WdJ critically revised the manuscript. HS conceptualized the study and critically revised the manuscript.

## Supplementary files

Supplementary file 1. GP Questionnaire.

Supplementary file 2. Referral Patterns.
