# Supplementary material for: Evaluating Cancer Care Networks; A Case Study of a Lung Cancer Care Network
Source: Int J Health Policy Manag. 2021 Sep 5;11(10):2103–14. doi: 10.34172/ijhpm.2021.98 (PMC9808273; doi:10.34172/ijhpm.2021.98)
Supplement: Supplementary file 1 — GP Questionnaire. [file ijhpm-11-2103-s001.pdf]

Article title: Evaluating Cancer Care Networks; A Case Study of a Lung Cancer Care Network

Journal name: International Journal of Health Policy and Management (IJHPM)

Authors' information: Anke Wind[1,2]*, René Limbeek[3], Henrike Bretveld[4], Robert van Schijndel[2], Daan Smits[5], Wouter de Jong[6], Hans Smit[1]

[1]Rijnstate, Arnhem, The Netherlands.

[2]Alliantie Regionale Topzorg (A.R.T.Z.), Arnhem, The Netherlands.

[3]Netherlands Comprehensive Cancer Organisation (IKNL), Utrecht, The Netherlands.

[4]Netherlands Cancer Registration, Netherlands Comprehensive Cancer Organisation (IKNL), Utrecht, The Netherlands.

[5]Slingeland Hospital, Doetinchem, The Netherlands.

[6]Ziekenhuis Gelderse Vallei, Ede, The Netherlands.

(*Corresponding author: ankewind@gmail.com)

Supplementary file 1. GP Questionnaire

Introduction:
In 2014, agreements were made between the hospitals in the Arnhem, Ede and Doetinchem region and Radiotherapy Group, location Arnhem, to better organize the care of patients with cancer. To this end, they are working together within the network. The network would like to know how healthcare professional experience the collaboration between the healthcare providers in the region. To this end, we are now looking specifically at lung cancer care and are interviewing both patients and care providers.
In doing so, we have also interviewed a patient from your practice, and this patient has given written permission to contact you as a general practitioner. In this questionnaire, we will ask you for your opinion about the relationship between you as a general practitioner and the hospital or different hospitals where the patient was treated.

y=yes
n=no
na=not applicable

1. Relationship GP - hospital (regarding this patient with lung cancer)
- Which hospital(s) did you have contact with? ....
- Were you informed in a **timely** manner by the hospital about
o The result of the diagnostic examination after referral? y/n/na
o The treatment plan after patient discussion in the MDO? y/n/na

o The results after completion of treatment?

Surgery y/n/na
Chemotherapy y/n/na
Chemoradiation y/n/na
Radiotherapy y/n/na
Immunotherapy y/n/na

o The patient's discharge from the hospital? y/n/nva

- Were you **fully** informed by the hospital about

o The result of the diagnostic examination after referral? y/n/na
o The treatment plan after patient discussion in the MDO? y/n/na
o The results after completion of treatment?

Surgery y/n/na
Chemotherapy y/n/na
Chemoradiation y/n/na
Radiotherapy y/n/na
Immunotherapy y/n/na

o The patient's discharge from the hospital? y/n/na

- Are you satisfied with the communication with the hospital(s)? y/n/na

o If not, what could be improved in your opinion?........................

2. Relationship HA - hospital (in general)

- Has the collaboration of the hospitals within A.R.T.Z. changed anything for you, positively or negatively? y/n

o If yes, what? ....

- Would you like to say anything else about the communication and agreements with the hospital / hospitals in general? y/n ....

3. Relationship GP - patient.

- Did you refer the patient for diagnostic testing after complaints? y/n
- At what stage of the disease process did you contact the patient? (multiple options possible)

o Immediately after diagnosis, before the start of the (1st) treatment y/n
o during the treatment phase y/n
o At the end of the treatment phase y/n
o After discharge from hospital y/n

- Did the contact match the patient's expectations? y/n

o If not, in what way not? ....

- At the time you contacted the patient, did you have sufficient information about his/her situation? y/n

o If not, what information was lacking?...........................

- What role do you see for yourself in the supervision of this patient? ....
- Would you like to say anything else about the communication with the patient?...........................
- Do you have any other comments or improvement points for the network?
