# Supplementary material for: Evaluating Cancer Care Networks; A Case Study of a Lung Cancer Care Network
Source: Int J Health Policy Manag. 2021 Sep 5;11(10):2103–14. doi: 10.34172/ijhpm.2021.98 (PMC9808273; doi:10.34172/ijhpm.2021.98)
Supplement: Supplementary file 2 — Referral Patterns. [file ijhpm-11-2103-s002.pdf]

Article title: Evaluating Cancer Care Networks; A Case Study of a Lung Cancer Care Network

Journal name: International Journal of Health Policy and Management (IJHPM)

Authors' information: Anke Wind[1,2]*, René Limbeek[3], Henrike Bretveld[4], Robert van Schijndel[2], Daan Smits[5], Wouter de Jong[6], Hans Smit[1]

[1]Rijnstate, Arnhem, The Netherlands.

[2]Alliantie Regionale Topzorg (A.R.T.Z.), Arnhem, The Netherlands.

[3]Netherlands Comprehensive Cancer Organisation (IKNL), Utrecht, The Netherlands.

[4]Netherlands Cancer Registration, Netherlands Comprehensive Cancer Organisation (IKNL), Utrecht, The Netherlands.

[5]Slingeland Hospital, Doetinchem, The Netherlands.

[6]Ziekenhuis Gelderse Vallei, Ede, The Netherlands.

(*Corresponding author: ankewind@gmail.com)

Supplementary file 2. Referral Patterns

2012-2013						
Network hospital (diagnosis)	Treatment in network		Referral to dedicated expertise center		Referral elsewhere	
	Number of patients	Percentage	Number of patients	Percentage	Number of patients	Percentage
Hospital A	590	97	8	1	9	1
Hospital B	326	94	11	3	10	3
Hospital C	233	96	6	2	4	2

Table 1a. Diagnostics and treatment within network, referral for treatment to dedicated expertise center or outside the region (first period, 2012-2013)

2014-2015						
Network hospital (diagnosis)	Treatment in network		Referral to dedicated expertise center		Referral elsewhere	
	Number of patients	Percentage	Number of patients	Percentage	Number of patients	Percentage
Hospital A	616	96	10	2	9	3
Hospital B	339	96	4	1	15	2
Hospital C	242	96	5	2	4	2

Table 1b. Diagnostics and treatment within network, referral for treatment to dedicated expertise center or outside the region (second period, 2014-2015)

2016-2017						
Network hospital (diagnosis)	Treatment in network		Referral to dedicated expertise center		Referral elsewhere	
	Number of patients	Percentage	Number of patients	Percentage	Number of patients	Percentage
Hospital A	584	97	1	0	6	1
Hospital B	327	96	14	2	13	4
Hospital C	292	97	2	1	8	3

Table 1c. Diagnostics and treatment within network, referral for treatment to dedicated expertise center or outside the region (third period, 2016-2017)
